# EDUCATION AND TRAINING I: COMPETENCY-BASED AND INTERGENERATIONAL APPROACHES

**DOI:** 10.1093/geroni/igae098.0171

**Published:** 2024-12-31

## Abstract

Competency-based training; Gerontology and Geriatrics training; Intergenerational programs and training

